# The Diagnostic and Clinical Utility of Autoantibodies in Systemic Vasculitis

**DOI:** 10.3390/antib8020031

**Published:** 2019-05-01

**Authors:** Elena Csernok

**Affiliations:** Department of Internal Medicine, Rheumatology and Immunology, Vasculitis-Center Tübingen-Kirchheim, Medius Klinik Kirchheim, University of Tübingen, 73230 Kirchheim-Teck, Germany; ecsernok@t-online.de

**Keywords:** autoantibodies, vasculitis, diagnostic and clinical significance, proteinase 3-/myeloperoxidase-ANCA, new recommendations

## Abstract

Considerable progress has been made in understanding the role of autoantibodies in systemic vasculitides (SV), and consequently testing for anti-neutrophil cytoplasmic antibodies (ANCA), anti-glomerular basement membrane antibodies (anti-GBM), and anti-C1q antibodies is helpful and necessary in the diagnosis, prognosis, and monitoring of small-vessel vasculitis. ANCA-directed proteinase 3 (PR3-) or myeloperoxidase (MPO-) are sensitive and specific serologic markers for ANCA-associated vasculitides (AAV), anti-GBM antibodies are highly specific for the patients with anti-GBM antibody disease (formerly Goodpasture’s syndrome), and autoantibodies to C1q are characteristic of hypocomlementemic urticarial vasculitis syndrome (HUVS; anti-C1q vasculitis). The results of a current EUVAS study have led to changes in the established strategy for the ANCA testing in small-vessel vasculitis. The revised 2017 international consensus recommendations for ANCA detection support the primary use PR3- and MPO-ANCA immunoassays without the categorical need for additional indirect immunofluorescence (IIF). Interestingly, the presence of PR3- and MPO-ANCA have led to the differentiation of distinct disease phenotype of AAV: PR3-ANCA-associated vasculitis (PR3-AAV), MPO-ANCA-associated vasculitis (MPO-AAV), and ANCA-negative vasculitis. Further studies on the role of these autoantibodies are required to better categorize and manage appropriately the patients with small-vessel vasculitis and to develop more targeted therapy.

## 1. Introduction

Primary systemic vasculitides (SV) are a clinical heterogeneous group of syndromes that are characterized by variable degrees of inflammation of the blood vessel wall. These severe and mostly rare disorders are difficult to diagnose, and the discovery that subgroups of systemic vasculitis are associated with autoantibodies against intracellular and tissue antigens has significant and prognostic implications on the diagnosis and management of the patients. Autoantibodies detected in primary systemic vasculitis are very heterogeneous with respect to their specificity and clinical significance. Only few of these autoantibodies are currently being used in routine practice in the diagnosis of patients with small-vessel vasculitis, such as anti-neutrophil cytoplasmic antibodies (ANCA) in ANCA-associated small-vessel vasculitis, anti-glomerular basement membrane (GBM) antibodies in anti-GBM antibody disease, and anti-C1q antibodies in immune complex-associated small-vessel vasculitis. This review will be focused on the recent advances in our understanding of the role of proteinase 3 (PR3-)-ANCA and myeloperoxidase (MPO-)-ANCA and other autoantibodies in the diagnostic workup and pathophysiology of patients with small-vessel vasculitis. The objectives of the review are to present and discuss the available new data on ANCA testing in vasculitis and to give advice on the clinical indications for ANCA diagnostics; to provide some data on the mechanisms of the development of ANCA and their pathogenetic role in vasculitis; to discuss the impact of ANCA antigen specificities on the classification of AAV; finally, to consider current data on the clinical and prognostic features of other autoantibodies detected in small-vessel vasculitis. 

## 2. Anti-Neutrophil Cytoplasmic Antibody (ANCA)

ANCA are important laboratory biomarker for so called “ANCA-associated vasculitides” (AAV). The AAV include granulomatosis with polyangiitis (GPA (formerly known as Wegener’s)), microscopic polyangiitis (MPA), and eosinophilic GPA (EGPA (formerly known as Churg Stauss syndrome)). Patients with GPA are predominantly PR3-ANCA-positive, whereas MPA and EGPA patients are typically associated with MPO-ANCA. 

ANCA were first described in the early 1980s as a cause of diffuse granular cytoplasmic immunofluorescence staining (C-ANCA) on ethanol-fixed neutrophils in association with glomerulonpehritis, vasculitis, and Wegener’s granulomatosis) [1,2]. In the last decades, ANCA has been detected in a variety of other disorders, including in patients with inflammatory bowel disease (IBD), autoimmune liver disease, connective tissue diseases, infections, and drug-induced vasculitis, often with multiple antigen specificities and unclear clinical significance ([3,4,5,6,7,8,9,10,11,12,13], for recent reviews on this topic, see Reference [14]). 

### 2.1. Significance of ANCA Target Antigens

There is a multitude of antigens recognized by ANCA. Although only two (PR3- and MPO-ANCA) have proven to be of clinical significance, ANCA directed against other antigens are sometimes mistakenly interpreted as clinically relevant. ANCA of specificity other than PR3 and MPO can be detected by ELISA screening for human neutrophil elastase (HNE-ANCA), lysosomal membrane protein-2 (LAMP-2-ANCA), bactericidal/permeability-increasing protein (BPI-ANCA), cathepsin G, lactoferrin, lysozyme, and other. Generally, these ANCA specificities have no diagnostic value for patients with AAV. 

However, one should be aware that some other ANCA antigen-specificities have also been suggested for consideration in the diagnostic workup of AAV. HNE-ANCA enables the differentiation between cocaine-induced midline destructive lesions and necrotizing midfacial lesions due to GPA [15,16]. Antibodies to lysosomal membrane protein-2 (LAMP-2) have been presented as a new ANCA subtype detectable in almost all necrotizing crescentic glomerulonephritis [17]. Although the results have been reproduced with different techniques in independent cohorts [18], this study could not be confirmed by others [19]. Thus far, anti-LAMP-2 antibodies have not been introduced into daily clinical practice. 

### 2.2. Methods of Detection 

Traditionally, indirect immunofluorescence test (IIF) on ethanol-fixed neutrophils is considered the reference method for ANCA detection; positive IIF results should always be followed by specific PR3- and MPO-ANCA immunoassays. Ideally, all three tests should be used in each sample [20,21]. Such diagnostic algorithm is based on an international consensus statement on ANCA testing issued in 1999 [20]. Since the establishment of this consensus many new antigen-specific immunoassays—such as fluoroenzyme immunoassay, chemiluminescence assay, and multiplexed flow immunoassay—have become available and this has challenged the position of IIF in the testing algorithm for ANCA in vasculitis [22,23]. 

In a recent EUVAS multicenter study, the diagnostic accuracy of a wide spectrum of novel technologies nowadays available for detection of PR3- and MPO-ANCA has been evaluated. For this study, diagnostic samples were included from AAV patients, i.e., GPA and MPA, as well as from patients suspected of, but eventually not having, AAV. The results are compared with two state-of-the-art ANCA IIF analyses, one based only on ethanol-fixed neutrophils and the other based on the combination of ethanol-fixed neutrophils, formalin-fixed neutrophils and HEp2 cells [24]. The dataset obtained has enabled us to draw a firm conclusion about the role of ANCA IIF in the diagnostic workup of AAV and was the basis of a novel international consensus on ANCA testing [24,25]. 

This new consensus was prepared by a EUVAS group of experts from four European laboratories. 

The recently published Revised 2017 International Consensus recommends that “high quality antigen-specific immunoassays can be used as a primary screening method for patients suspected of having the ANCA-associated vasculitides GPA and MPA without categorical need for IIF” [26]. Furthermore, it was shown that appropriately designed reference ranges for antibody levels improve interpretation of antigen-specific immuno-assays [27]. The comparison with the EUVAS study of Hagen et al., which has paved the way for the first international consensus on detection of ANCA, showed that the diagnostic performance (sensitivity and specificity) of ANCA immunoassays has significantly improved [25,28].

In case of a high clinical suspicion of vasculitis and a negative test result in PR3- and MPO-ANCA immunoassay, the revised international consensus on ANCA testing recommend performing IIF or a second antigen-specific immunoassay. Performing a second assay or IIF can also increase the specificity in case of low positive test results [26]. It has furthermore been highlighted that the ANCA test should be used in patients with a high index of clinical suspicion of small-vessel vasculitis [26].

### 2.3. Clinical Utility

Today, testing for ANCA has become part of routine labor investigation of patients with suspected necrotizing vasculitis or/and idiopathic glomerulonephritis. However, a number of studies have demonstrated that a test for ANCA, as currently ordered, is not useful to identify AAV patients when ANCA testing is used in non-selected hospitalized patients [29]. Although PR3- and MPO-ANCA is very sensitive for AAV, positive ANCA are detected in many other inflammatory conditions. Therefore, ANCA have low PPV for AAV in unselected populations. AAV are very rare disorders and application of ANCA test in an unselected population results in a high number of “false” positives. Clinical ANCA test-ordering guidelines have been proposed [20], and the evaluation of these test-ordering guidelines has demonstrated that the ordering of ANCA test in patients with high suspicion of vasculitis reduces “false” positive results and a significant cost savings can be made [30,31]. Interestingly, Arnold et al. investigated the impact of a “gating policy” at a single regional center in the year prior to and following the consensus guidelines, and demonstrated that adherence to a “gating policy” for ANCA testing coupled with close liaison between clinician and laboratory does not result in either a missed or delayed diagnosis of AAV [32]. 

The highest specificity of ANCA in vasculitis is obtained by a combination of IIF and antigen-specific immunoassays. With this strategy, PR3- or MPO-ANCA is detectable in nearly all patients with active generalized GPA or MPA, but only in approximately 60% of patients with limited (“initial phase”) disease. However, among patients with EGPA, the prevalence of ANCA may be as low as 40% [3,4]. 

A positive ANCA result, as it is today ordered by practicing clinicians, is not a definitive diagnostic indicator of AAV. Patients with ANCA should receive a careful workup, because conditions other than vasculitis, such as infections or drug abuse, may be implicated in the presence of ANCA, and may be dramatically worsened by immunosuppression. Whereas the diagnostic utility of ANCA as a biomarker of AAV is very high in the right clinical context, their diagnostic value in non-vasculitis conditions is very limited. Thus, a positive result on antigen-specific immunoassays ANCA test support the diagnostic of AAV but cannot supplant the need for critical appraisal of all findings including the patient history, clinical and imaging, as well as other laboratory results. Moreover, histological tissue diagnosis remains the gold standard and should be sought in every patient. 

The utility of ANCA for monitoring disease activity and guidance of treatment decisions in AAV has been long-debated. In a meta-analysis study, it was found that the increase or the persistence of ANCA in patients who achieved remission is not strongly associated with relapses [33]. These observations led to the conclusion that the value of serial measurements of ANCA in patients in remission is limited [20]. In recent studies, two groups have demonstrated that the serial testing of ANCA to monitor disease activity may be useful in prediction of renal and/or pulmonary small-vessel vasculitis relapse, but not for the granulomatous inflammation [34,35]. Kemna et al. found that ANCA rises correlated with relapses in AAV patients who presented with renal involvement (hazard ratio HR, 11.9; 95% confidence of interval CI, 5.01 to 24.55), whereas relapses in patients with non-renal disease were weekly associated with rises in ANCA values (HR, 2.79; 95% CI, 1.30 to 5.87) [34]. A study by Fussner and colleagues evaluated the serial ANCA measurements in the Rituximab versus cyclophosphamide for ANCA-associated vasculitis (RAVE) trial [35]. Although the authors found that rises in ANCA titers during follow-up of AAV patients is not a very sensitive or specific predictor of relapse in general, they observed that the rise of PR3-ANCA level during complete remission conveys an increased risk of relapse among patients with renal vasculitis (HR 7.94) or alveolar hemorrhage (HR 24.19) and those treated with rituximab (but not those treated with cyclophosphamide and azathioprine). The authors suggested that serial measurements of PR3-ANCA may be informative in these disease phenotypes, but the risk of relapse must be weighed carefully against the risks associated with therapy [35]. 

Concerning the role of ANCA as biomarker for choice of treatment, in a recent subsidiary RAVE study, Unizony et al. showed that PR3-AAV patients respond better to rituximab than the cyclophosphamide- and azathioprine-treated patients [36]. The authors suggest that PR3-ANCA may be used to guide immunosuppressive therapy in AAV [36]. However, the EULAR recommendations for management of AAV state that treatment decisions during patient follow-up should rather be based on clinical evaluation and not on changes of the ANCA levels alone [37].

### 2.4. Pathogenic Potential of ANCA in Small-Vessel Vasculitis

Determining the mechanisms by which ANCA develops and shapes the immune response in AAV is not completely understood. There is a great deal of evidence that certain environmental exposures (e.g., silica), infectious pathogens (e.g., Staphylococcus aureus), or drugs (e.g., cocaine) are associated with AAV, but there is no high attributable risk of disease from any specific agent. The postulated mechanisms by which infections may trigger generation of ANCA include autoantigen complementarity, epigenetic silencing, molecular mimicry, neutrophil extracellular traps (NETs) formation, and NETosis. Current data demonstrated that NET-associated autoantigens initiate generation of ANCA, and persistent NET exposure at sites of inflammation could further augment established pathogenic ANCA production and promote the autoimmune response [38,39,40]. Furthermore, in a recent genome-wide association study (GWAS) of AAV, it was confirmed that patients with GPA and MPA have a genetic predisposition to developing ANCA and the disease [41]. The strongest genetic associations were with ANCA serotype, not with clinical phenotype (GPA vs. MPA). PR3-ANCA positive patients were associated with *HLA-DP,* genes encoding PR3 *(PRTN3),* and their main inhibitor alpha1-anti-trypsin *(SERPIN1).* In contrast, MPO-ANCA-positive patients were associated with *HLA-DQ* [41]. Even though it is currently unclear why patients make ANCA, what makes PR3 and MPO so unique among all the described ANCA target antigens is that only ANCA with these two molecules is associated with small-vessel vasculitis. 

For more than three decades, PR3- and MPO-ANCA were believed to play a central role in the development of necrotizing vasculitis and glomerulonephritis, but the mechanism whereby they contribute to damage of vessel walls is only partially understood.

The current concept of ANCA-induced vascular damage was mainly developed from in vitro studies and is supported by the data from clinical investigations and in vivo experimental animal models. The most accepted model of ANCA-induced vasculitis proposes that ANCA activate primed neutrophils, and full activated neutrophils damage the endothelium, leading to an escalation of inflammation that culminates in necrotizing vasculitis [42]. 

Recently, Schreiber et al. have identified a mechanistic link between ANCA-induced neutrophil activation, regulated necrosis (necroptosis), generation of NETs, activation of complement pathway, and endothelial cell damage with consecutive vasculitis and necrotizing glomerulonephritis in AAV [43]. The authors used pharmacologic and genetic approaches in murine disease models and showed that NETS were formed in response to MPO-ANCA, and that ANCA-induced NET generation is controlled by mediators of necroptosis pathway (RIPK1/3 and MLKL) [43]. Moreover, it was demonstrated that the inhibition of necroptosis-induced kinases completely prevents ANCA vasculitis. The authors suggest that necroptosis pathway molecules such as RIPK1 may represent novel therapeutic strategy in AAV [43].

It is interesting to note that the recent studies highlight the inflammatory role of PR3 and have shown that the unique structural and functional characteristics of this molecule might be key contributors to the systemic inflammation and to the immune dysregulation in PR3-AAV [44].

In summary, recent studies investigating the potential pathogenic role of ANCA suggest, but do not definitively prove, that ANCA are directly pathogenic. However, all of these publications clearly show that ANCA, in combination with exogenous factors, are able to aggravate the clinical inflammatory process and may result in systemic vasculitis and glomerulonephritis.

### 2.5. The Role of ANCA Antigen Specificity in the Classification of Small Vessel Vasculitis 

Many attempts have been made to classify the vasculitis syndromes and a major breakthrough was made in the last years, when several groups discovered that ANCA specificity could be better than clinical diagnosis for defining groups of patients. These studies show that PR3-ANCA-positive patients differ from MPO-ANCA-positive patients with respect to genetic basis, epidemiology, clinical manifestations, histological findings, response to therapy, and pathogenesis. The use of ANCA serotypes for disease classification provides immediate diagnosis based on the presence of PR3- and/or MPO-ANCA. It was demonstrated that ANCA serotyping distinguishes distinct classes of ANCA disease: PR3-ANCA-associated vasculitis (PR3-AAV), MPO-ANCA-associated vasculitis (MPO-AAV), and ANCA-negative vasculitis (reviewed by Reference [45]). 

The first genome-wide association study provides an important step forward in the classification of AAV. The susceptibility genes statistically significant associated with PR3- or MPO-ANCA patients were mainly identified (see above), suggesting that they are dealing with two different disorders [41]. Clinical manifestations differ between PR3-AAV and MPO-AAV. It was found that extra-renal organ manifestations, granulomatous inflammation, and a higher relapse rate are more frequent in patients with PR3-AAV [46,47]. In contrast, MPO-AAV patients have more frequent kidney-limited disease, display more severe renal scarring, and a worse renal prognosis (HR 2.1 95% CI 1.11–3.80) [48]. Furthermore, patients with MPO-ANCAs are more likely to have renal pathology classified as mixed or sclerotic, and to have a strong association with lung fibrosis compared to patients with PR3-ANCAs [49,50]. Moreover, many studies have shown that PR3-AAV differs from MPO-AAV with respect to treatment response, relapse rate, and outcome [51,52]. 

Taken together, the classification of patients by ANCA specificity (PR3- vs. MPO-ANCA) provides practical diagnostic criteria better aligned to patient phenotype, outcomes, and treatment responses than does their classification by clinical diagnosis (GPA vs. MPA and EGPA) (for a review, see Reference [45]). 

## 3. Anti-Glomerular Basement Membrane Antibodies 

Anti-GBM antibodies are very specific biomarkers for patients with anti-GBM antibody disease (formerly Goodpasture’s syndrome) which has been recently classified as immune complex vasculitis affecting glomerular and/or pulmonary capillaries [53]. Approximatively one-third of patients with pulmonary-renal syndromes are anti-GBM antibodies positive. The non-collagenous domain 1 (NC1 domain) of the alpha3-chain of type IV collagen is the main target antigen, and only autoantibodies to this epitope correlate with disease activity and can induce disease in animal model [54]. These autoantibodies are strongly associated with rapidly progressive glomerulonephritis and their association to lung hemorrhage is less pronounced.

Preferred method to detected and quantify circulating anti-GBM antibodies is ELISA using purified NC1 domain of type IV collagen, which showed the highest sensitivity [55]. The sensitivity of IIF is moderate, and this method can give false positive results in cases of diabetes and in biopsy from renal transplants. 

In many patients with anti-GBM antibody disease (20%–30%), it is not uncommon to find anti-GBM antibodies co-occurring with MPO-ANCA. These two autoantibodies are found in similar settings and often in the same patients.

A pathogenetic role for anti-GBM antibodies has been demonstrated [56]. Numerous animal models have been described and transfer of anti-GBM antibodies alone can induce disease but always with a mild glomerulonephritis (GN) [57,58,59]. Furthermore, there is evidence for T-cell component in anti-GBM disease. Recently, a nephritogenic T cell epitope (P14 a3127-148) on human Goodpasture antigen was identified and this epitope not only induced severe anti-GBM nephritis, but also initiated epitope spreading in WKY rats [60].

## 4. Anti-C1q Antibodies

Anti-C1q antibodies are strongly associated with immune complexed mediated diseases, especially HUVS (anti-C1q vasculitis), recently classified as an immune complex small-vessel vasculitis (53). These autoantibodies are diagnostic marker for HUVS since they occur in 100% of patients. There is a correlation between C1q antibodies and renal involvement glomerulonephritis and according to development of nephritis. These autoantibodies are found in low frequency in other SV such as anti-GBM nephritis and Behcet’s disease with vascular involvement [61]. 

The collagen-like region (CLR) of the subunit of complement factor C1q is the target of this autoantibody. The major epitope is a linear 13-mer peptide GRPGRRGRPGLKG of the A chain of C1q. Circulating anti-C1q antibodies are detected by using ELISA with intact C1q, or CLR or by using the 13-mer peptide GRPGRRGRPGLKG [62]. Detection of these autoantibodies is indicated to establish the diagnosis of HUVS and for differential diagnosis of GN. 

The pathophysiology of HUVS has not been fully clarified, and a pathogenetic role for anti-C1q antibodies was proposed [63]. However, to date, the pathogenetic role of these autoantibodies on HUVS has not been studied in detail. Today, there is much evidence that these autoantibodies play a part in the pathogenesis of systemic lupus erythematosus (SLE), and especially in the development of nephritis, a major complication of disease. In a recent study, it was investigated how anti-C1q antibodies contribute to the development of nephritis in mouse models [64]. The authors showed that anti-C1q antibodies can be pathogenic to the kidney only in the context of C1q-containing glomerular immune complexes (C1q-fixing anti-GBM antibodies) [64]. However, the patients with HUVS are unrelated to renal pathology. 

## 5. Anti-Endothelial Cell Antibodies

The autoantibodies that are specifically directed to endothelial cells antigens (AECA) have been detected in a heterogeneous group of patients with SV (AAV, Kawasaki’s disease, IgA vasculitis, and Behcet’s disease). Some findings suggest that AECA may be involved in pathogenesis of vasculitis and can be used as biomarkers of disease activity in the respective disease. However, these autoantibodies are not specific for any particular disease. 

The autoantigens involved in AECA reactivity in SV are very heterogeneous and diverse. Recently, the target antigens of AECA in AAV patients have been investigated by using a proteomic approach, and the proteins identified as target antigens were lamin A, vimentin, alpha-enolase, far upstream binding protein 2, and protein disulfide-isomerase A3 precursor [65]. 

Several methodological approaches have been established for detection of AECA, but currently, specific and highly sensitive immunoassays are not available for routine use. Furthermore, AECA detection methodology needs to be standardized, and the question still remains whether AECA determination should be used in the evaluation of vasculitis. 

## 6. Other Autoantibodies

Recently, it was reported that a subset of PR3-ANCA-positive patients (approx. 25%) harbor antibodies against human plasminogen [66]. The presence of antibodies recognizing plasminogen, a key component in the fibrinolytic system, correlated with venous thromboembolic events (VTEs) [66]. Berden et al. have shown that anti-plasminogen antibodies, particularly in combination with antibodies against tissue plasminogen activator, compromise fibrinolysis and correlates with hallmark renal histologic lesions and reduced renal functions [67]. 

Other autoantibodies such as anti-alpha-enolase antibodies, antiphospholipid antibodies, antinuclear antibodies, anti-citrullinated protein/peptide antibodies, anti-ferritin, anti-laminin, rheumatoid factors, etc., are commonly found in sera of patients with SV and further are needed to determine the usefulness of these autoantibodies in the clinical practice. 

## 7. Conclusions

Over the last decades, considerable progress has made in detection and characterization of novel autoantibodies in small-vessel vasculitis. It was demonstrated that the occurrence of a particular profile of autoantibodies in a patient can be associated with a defined type of clinical conditions. In particular, in the ANCA diagnostic novel international consensus for ANCA, testing in GPA and MPA was established. These guidelines state that antigen-specific immunoassays are the preferred approach to detect ANCA for diagnosis of AAV. However, from the data presented here, the role of autoantibody for the identification of disease phenotypes and to tailor treatment requires further studies. 
